# The Rubber Syringe as a Substitute for the Stomach Pump

**Published:** 1881-02

**Authors:** 


The Rubber Syringe as a Substitute for the Stomach Pump.—Dr. J. A. Grant reports in the *Canada Medical and Surgical Journal* a case in which a child had swallowed a quantity of carbolic acid bug poison. After administering a quantity of oil the doctor found that his stomach pump was broken.

Having no better appliance at hand he inserted the long tube of an ordinary india-rubber injection pipe into the stomach quite easily, and pumped in a quantity of water, which he hoped to remove by leaving the tube *in situ* and changing the position of the child. Such, however, was unnecessary, as after the tube was detached from the body or central part of the instrument the fluid from the stomach rushed out at a bound through the tube, and thus the contents of the stomach were rapidly and unexpectedly removed. This washing out process was repeated three times in the space of a few minutes, after which the ejected fluid gave no particular indications of carbolic acid. After being two hours in charge of the case, milk and lime water were administered freely, which for several hours was swallowed with considerable difficulty. The return of sensibility was slow but gradual, and for fully five hours the body temperature was low, pupils moderately contracted, skin pale, and the lips, chin and mucous membrane of the mouth presented quite an excoriated appearance, from the direct effects of the carbolic acid, all of which was much benefitted by the free application of olive oil.

On the third day from the date of the accident the child appeared as well as could possibly be expected under the circumstances, and only at times complaining of a sense of gastric discomfort, and more or less irritability of temper, all of which had almost entirely subsided within the first week. As is well known, the employment of an ordinary stomach pump requires care, in order to avoid injury to the soft parts from the ivory end of the tube. Several such records of injury are published. The application of the ordinary india-rubber syringe is, however, superior to any other appliance in such cases—first, from its great simplicity; second, from the ease of application, and third, from the fact that when the body of the instrument is detached, the ordinary contraction of stomach, diaphragm and abdominal muscles will expel the contents of the stomach freely, without any effort being made to pump out the fluid in the usual way. Thus it appears that the contact of the tube with the gastric walls excites reflex expulsion, like the tickling of the throat with the finger or a feather in order to induce emesis. Thus we have an inexpensive, simple and practical means of removing poisonous substances from the stomach, and with a degree of rapidity vastly superior to any other appliance heretofore recommended under such trying circumstances. From an examination of the ejected fluid, the quantity of carbolic acid was about a tablespoonful, the ill effect of which on the gastric mucous membrane was considerably modified from the fact that the stomach contained several ounces of fluid at the time of the accident.

